# 1,4-Disubstituted 1,2,3-Triazoles as Amide Bond Surrogates for the Stabilisation of Linear Peptides with Biological Activity

**DOI:** 10.3390/molecules25163576

**Published:** 2020-08-06

**Authors:** Lisa-Maria Rečnik, Wolfgang Kandioller, Thomas L. Mindt

**Affiliations:** 1 Ludwig Boltzmann Institute Applied Diagnostics, General Hospital Vienna, 1090 Vienna, Austria; lisa.recnik@lbiad.lbg.ac.at; 2 Institute of Inorganic Chemistry, Faculty of Chemistry, University of Vienna, 1090 Vienna, Austria; wolfgang.kandioller@univie.ac.at; 3 Department of Biomedical Imaging and Image Guided Therapy, Division of Nuclear Medicine, Medical University of Vienna, 1090 Vienna, Austria

**Keywords:** 1,4-disubstituted 1,2,3-triazoles, CuAAC, peptidomimetics, amide bond surrogate, metabolic stabilisation

## Abstract

Peptides represent an important class of biologically active molecules with high potential for the development of diagnostic and therapeutic agents due to their structural diversity, favourable pharmacokinetic properties, and synthetic availability. However, the widespread use of peptides and conjugates thereof in clinical applications can be hampered by their low stability in vivo due to rapid degradation by endogenous proteases. A promising approach to circumvent this potential limitation includes the substitution of metabolically labile amide bonds in the peptide backbone by stable isosteric amide bond mimetics. In this review, we focus on the incorporation of 1,4-disubstituted 1,2,3-triazoles as amide bond surrogates in linear peptides with the aim to increase their stability without impacting their biological function(s). We highlight the properties of this heterocycle as a *trans*-amide bond surrogate and summarise approaches for the synthesis of triazole-containing peptidomimetics via the Cu(I)-catalysed azide-alkyne cycloaddition (CuAAC). The impacts of the incorporation of triazoles in the backbone of diverse peptides on their biological properties such as, e.g., blood serum stability and affinity as well as selectivity towards their respective molecular target(s) are discussed.

## 1. Introduction

Peptides represent one of the major classes of biomolecules used as diagnostics and therapeutics. [1] Since the introduction of solid-phase peptide synthesis (SPPS) by Merrifield [2], which allows for a quick, easy and automatable synthesis of peptides, their pharmaceutical impact grew enormously due to their high specificity and low off-target side-effects. However, applications of peptides in medicine can be limited due to their low in vivo stability [3]. They are susceptible to rapid degradation by proteases via hydrolysis of the amide bonds in their backbone structure [4]. This leads to a poor oral bioavailability due to the presence of proteases in the digestive system and a low metabolic stability in vivo derived from the degradation in the blood plasma mediated by soluble and membrane-bound peptidases [5,6].

Therefore, substantial effort has been put into the development of stabilisation techniques for metabolically labile peptides. Different approaches have been studied involving structural variations of the peptide sequence. For example, modification of the C- or N-terminus of a peptide, cyclisation of linear peptides or incorporation of d-amino acids and other unnatural amino acids in the sequence have been shown to have a positive impact on peptide stability in blood serum [3,5,7]. Another approach is the coadministration of protease inhibitors to impede fast degradation and enhance in vivo blood serum stability [5,8].

**Figure 1:**
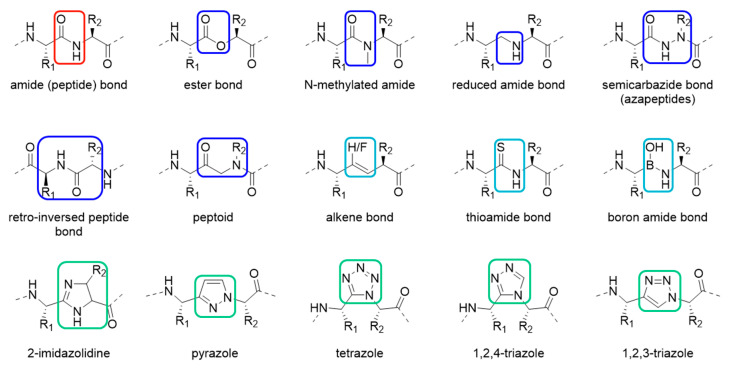
Various reported mimetics of a peptide bond (top left, red) including common motifs (blue), motifs containing heteroatoms (light blue) or heterocycles (green).

In order to prevent degradation by proteases, amide bonds susceptible to cleavage can be substituted with isosteric amide bond surrogates. [9] Those peptidomimetics resemble native peptides but contain synthetic non-peptidic structural features to improve the therapeutic or diagnostic properties of the molecule. The most common peptide bond mimetics include esters, *N*-methylated amide bonds, reduced amide bonds, semicarbazides, retro-inversed peptide bonds, peptoids and alkenes (Figure 1) [9,10]. Introduction of heteroatoms such as sulfur, fluorine or boron lead to further variation [11,12,13,14,15]. The incorporation of heterocycles in the peptide backbone is also reported. [16,17] The first example of an aromatic heterocyclic amide bond mimetic in literature is the substitution of the amide bond with 2-imidazolidine [18]. Other well-studied heterocyclic surrogates are pyrazoles, tetrazoles and 1,2,4-triazoles and many more examples can be found in the literature [19,20,21]. One of the most recently emerged amide bond mimetics in peptides is the 1,2,3-triazole scaffold [22].

In medicinal chemistry, the 1,2,3-triazole motif is frequently used for the replacement of amide bonds and their application in small molecules has already been highlighted elsewhere [23,24]. In 2003, the first report of a cyclic peptidomimetic was published where a 1,4-substituted 1,2,3-triazole was used as an isostere for the amide bond to obtain molecules which self-assemble into nanotubes [25]. In the following years, several groups investigated the structural consequences of the incorporation of one or more triazoles on the secondary structure of resulting peptidomimetics such as α-helices and β-sheets [26,27,28,29,30,31,32,33]. Another focus of attention was the synthesis and biological evaluation of cyclic peptidomimetics with one or more triazoles in the backbone [34,35,36,37,38,39,40,41,42,43]. In most cases, the formation of the triazole ring was used as a strategy for the (macro)cyclisation of the peptidomimetic. In a similar manner, CuAAC was also applied for the synthesis of large peptides by ligating smaller fragments [44]. A handful of studies investigated the conformation of a dipeptide with the central amide bond being substituted by a triazole [45,46,47].

In contrast to a metabolically labile amide bond, 1,2,3-triazoles are not cleaved by proteases. Hence the substitution of a peptide bond by a triazole (Ψ[Tz], a so-called amide-to-triazole switch) represents a promising strategy to increase the peptide’s in vivo stability. This property is not only important for the therapeutic efficacy of a drug but also for diagnostic means [48]. A number of examples of triazolo-peptidomimetics included in this review concern their application as radiopharmaceuticals. In nuclear medicine, small radiolabelled peptides are the agents of choice for tumour-targeting vectors in diagnostic imaging of tumour entities. For example, the peptide can be conjugated via its N- or C-terminus or a sidechain to a chelator that can stably complex a metallic radionuclide such as lutetium-177 or gallium-68 [48]. The radiopharmaceutical is applied intravenously and then distributes in the entire body. It specifically binds to the cancer cells due to high receptor affinity and after internalisation, it gets accumulated inside the tumour. This leads to an increased concentration of radioactivity inside the target, which is then detected by either single photon emission computed tomography (SPECT) or positron emission tomography (PET) depending on the employed radionuclide [48,49]. In the field of SPECT- and PET-imaging, peptides are preferred over antibodies as tumour-targeting vectors because of their favourable pharmacokinetic properties, their synthetic availability and their low cost [50]. In general, their rapid distribution, fast tissue penetration and quick blood clearance enable imaging with higher resolution and after shorter incubation times compared to antibodies. Furthermore, healthy tissue is less exposed to radiation which makes radiolabelled peptides a safer option for treatment of patients [50,51]. The abundance of regulatory and other peptides, which display high affinity for receptors that are often massively overexpressed in cancer cells, constitutes a vast pool of possibilities for the development of diagnostic tools. However, one limitation is their short biological half-life in blood plasma due to proteolysis. The number of approved radiolabelled peptides for imaging or therapy is still low partly due to low in vivo stability. Currently a great effort is ongoing to improve their stability whilst maintaining (or even increasing) the affinity to the receptor [48]. Besides their use as radiopharmaceuticals, other applications of triazolo-peptidomimetics include endogenous peptides involved in signalling, protease inhibitors and inhibitors of protein-protein interaction and will be discussed in detail in the following.

In this review, we highlight the properties of 1,2,3-triazoles as *trans*-amide bond isosteres and the synthesis of triazolo-peptidomimetics. We discuss the application of the amide-to-triazole switch in peptides with the aim to improve their stability. We limit our review to the modification of linear, high affinity peptides which exhibit a known biological function and where one or more amide bonds are replaced with a 1,4-disubstituted 1,2,3-triazole. The use of 1,5-disubstituted 1,2,3-triazoles as surrogates for *cis*-amide bonds is not within the scope of this review, but can be found summarised elsewhere [42,52,53,54,55,56]. This review is organised by peptides and listed in chronological order.

## 2. 1,2,3-Triazole as Amide Bond Isostere

**Figure 2:**
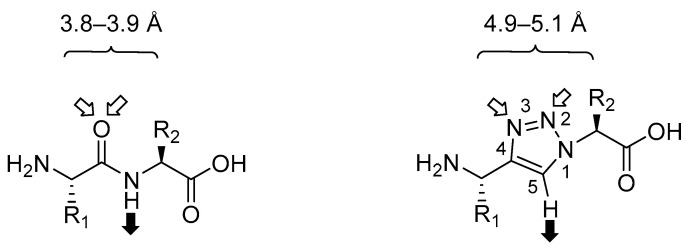
Comparison of an amide bond (left) and an 1,4-disubstituted 1,2,3-triazole (right). Distance between two residual side chains given. White arrows indicate H-bond receptors, black arrow H-bond donors. Modified from Valverde et al. [22].

1,4-Disubstituted 1,2,3-triazoles are excellent isosteres for the *trans*-amide bond as they mimic the electronic properties such as dipole moment and hydrogen-bond properties of the amide bond in contrast to many other surrogates (Figure 2) [22]. The dipole moment of the triazole moiety is slightly higher than in amide bonds (~4.5 Debye vs. ~3.5 Debye) which allows for the polarisation of the proton at C-4 so that it can serve as H-bond donor similar to the NH in amides [57,58]. The lone pairs at *N*-2 and *N*-3 serve as weak H-bond acceptors, hence well mimicking the amide bond. [59,60] Because of the high dipole moment, the triazole unit could align with that of other amide bonds to stabilise the secondary structure of peptides [61]. Furthermore, the aromatic heterocycle is able to form intra- or intermolecular π-interactions [62]. When comparing the structure and the size of the two units, the triazole surrogate leads to a slightly longer distance (4.9–5.1 Å) between the two neighbouring amino acid residues than the amide bond (3.8–3.9 Å), which is thus more similar to the distance encountered in peptides incorporating β-amino acids (4.9 Å) [22,23,63]. The heterocycle reproduces the planarity of the amide bond, although it also displays a higher rigidity due to the aromatic structure. All these similar features make this heterocyclic motif a good amide bond mimetic, which is highly stable to enzymatic cleavage by proteases, acid or base hydrolysis and reductive or oxidative conditions [64]. However, due to this high chemical stability, it is still unknown how triazoles are metabolised and further investigations are needed to determine their biological fate [61].

## 3. Synthesis of Triazolo-Peptidomimetics

**Figure 3:**
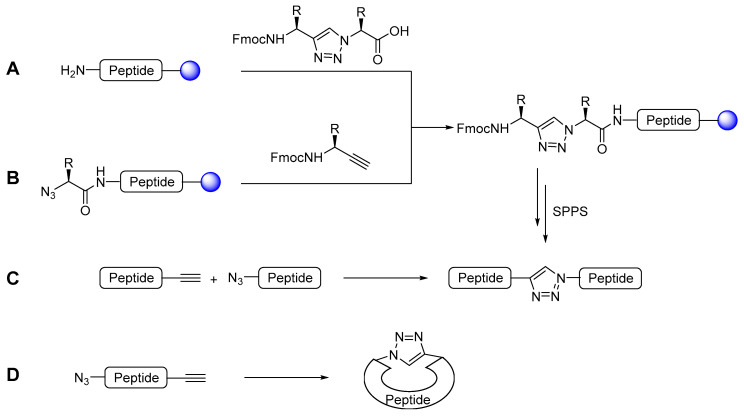
Schematic representation of the different strategies for the synthesis of triazolo-peptidomimetics: **A**: Formation of triazole-containing dipeptide in solution prior to coupling onto solid-phase synthesis. **B**: Formation of triazole on solid-phase. **C**: Triazole formation as ligation step of two peptide fragments. **D**: Triazole formation as cyclisation strategy. Modified from Valverde et al. [22].

In recent years, the regioselective synthesis of 1,2,3-triazoles became easily accessible. In 2002, the groups of Meldal and Sharpless reported independently the Cu(I)-catalysed azide-alkyne cycloaddition reaction (CuAAC) which leads to the regioselective formation of 1,4-disubstituted 1,2,3-triazoles under mild conditions [65,66]. This reaction is classified as a click chemistry reaction as it is high yielding, wide in scope, simple to perform, produces no (or easily removable) side-products and can be conducted in a wide range of solvents [67]. Due to this outstanding versatility, this reaction quickly had a high impact in many areas of chemistry. Applications of the CuAAC range from organic and medicinal chemistry to material science and biological chemistry [68,69,70,71,72]. This reaction is also a versatile tool for peptide chemists, either for cyclisation reactions (head-to-tail, head/tail-to-sidechain or sidechain-to-sidechain) or cyclodimerisations, as disulfide bridge replacement, for ligation of peptide fragments or bioconjugation to other (bio)molecules [73,74]. Meldal reported the use of CuAAC on solid phase which further enhanced its use in peptide synthesis [65]. The introduction of the triazole motif into the backbone of the peptide can be easily performed in solution or on solid phase as depicted in Figure 3. 

The CuAAC can be performed in solution between an *N*-protected α-amino alkyne and an α-azido carboxylic acid leading to a triazole-containing dipeptoid building block which can then be employed for SPPS (pathway A). Alternatively, the CuAAC can be carried out directly on solid phase by transforming the immobilised, deprotected N-terminal amine on-resin into an azide and subsequently reacting it with a protected α-amino alkyne together with a Cu(I) salt (or a source thereof) for the CuAAC (pathway B). CuAAC can also be envisaged for the ligation of two larger peptide fragments with an azide- and an alkyne-terminus, respectively (pathway C). In case of cyclic triazole-containing peptides, the triazole formation may be used as the cyclisation step after the synthesis of the linear peptide sequence bearing an azide and an alkyne unit (pathway D).

**Figure 4:**
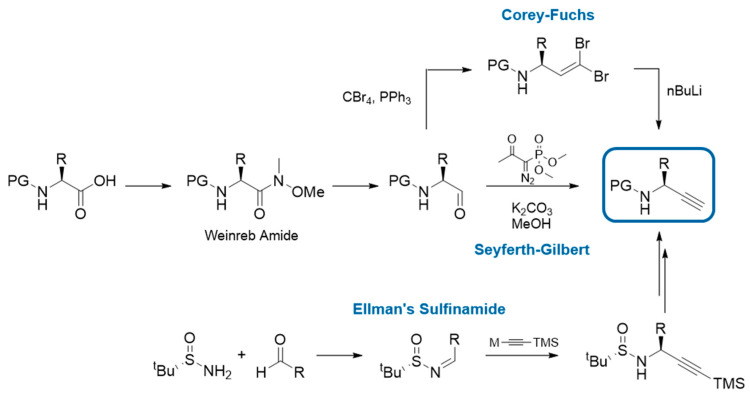
Different approaches for the synthesis of α-amino alkynes: The most common synthesis is starting from an amino aldehyde via a Seyferth-Gilbert homologation using the Bestmann-Ohira reagent. Another possibility from the same starting material is the Corey-Fuchs reaction. An alternative approach is the condensation of an achiral aldehyde and Ellman’s auxilliary followed by the conjugate addition of an organometallic alkyne reagent to the sulfinamide. M = Metal (e.g., Li or MgX), PG = protecting group, R = amino acid side chain, TMS = trimethylsilyl.

In solid phase synthesis, the triazole moiety is incorporated into the peptide backbone in two steps. First, the free amine of the elongated peptide chain is transformed into an azide via a diazotransfer reaction. Imidazole-1-sulfonyl azide hydrochloride (ISA٠HCl) is a relatively safe and stable diazotransfer reagent [75] and the reaction can be carried out at room temperature in solvents compatible with solid phase synthesis (e.g., DMF). Alternatively, chiral α-azido acids are also commercially available and can be used directly for SPPS. The second step consists of the CuAAC where the terminal azide is reacted with an Fmoc-protected α-amino alkyne under Cu(I) catalysis employing different sources of the metal (i.e., CuI, CuBr∙Me_2_S, Cu(CH_3_CN)]PF_6_ or CuSO_4_·5H_2_O with sodium ascorbate) [76,77,78,79]. α-Amino alkynes are not commercially available yet, however, their synthesis can be achieved in a few steps from Fmoc-protected amino acids (Figure 4). The most common and convenient method involves the formation of the corresponding α-amino aldehyde via the Weinreb amide and the subsequent Seyferth-Gilbert homologation using the Bestman-Ohira reagent [80]. Besides, the Corey-Fuchs reaction for the transformation of aldehydes to alkynes can be employed for the synthesis of Boc-protected α-amino alkynes [81]. However, it is known that this approach may lead to partial racemisation of the α-amino alkyne [52,56,82]. The use of α-amino alkynes as a mixture of enantiomers results in the formation of diastereomers when used for peptide synthesis and additional measures (i.e., purification of the alkynes using chiral HPLC or separation of the resulting diastereomeric peptide products by HPLC) have to be taken into account. An alternative synthesis of α-amino alkynes involves the condensation of Ellman’s auxiliary onto aldehydes and subsequent conjugate addition of an organometallic reagent to the resulting sulfinamide (Figure 4) [56]. However, this method is limited to amino acids without acid-sensitive protecting groups in their side chains.

Reaction times for the CuAAC on solid phase are generally longer than for Fmoc-SPPS peptide couplings. It is usually performed at room temperature overnight and no heating or microwave reactor is necessary. CuAAC on solid phase results in quantitative conversion which can be followed by the Punna-Finn test, a colorimetric test developed for the detection of aliphatic azides similar to the Kaiser test [83]. The CuAAC can also be used to ligate several peptide fragments as an alternative cyclisation approach for peptides.

## 4. Applications

### 4.1. PACE4 Inhibitors

Paired basic amino acid cleaving enzyme 4 (PACE4, also known as proprotein convertase subtilisin/kexin type 6 PCSK6) belongs to the family of pro-protein convertases and plays an important role in tumour aggressiveness. A patent from Dory and co-workers describes the development of more stable and selective PACE4 inhibitors for treating a variety of different cancers based on the octapeptide multi-Leu peptide [84]. Various modifications were introduced in this peptide including the replacement of amide bonds with a 1,2,3-triazole and in silico analyses were performed to select potent inhibitors. Amongst them were the two triazole-containing peptidomimetics **P1** and **P2** with a triazole in positions Leu^2^-Leu^3^ and Leu^1^-Leu^2^, respectively (Table 1). However, the synthesis of these two compounds is not described. Half-life (t_1/2_) in blood plasma and the inhibitor constant (K_i_) was determined. Interestingly, compound **P1** displayed a two-fold lower half-life in blood plasma and a decreased potency (t_1/2_ = 1.0 h, K_i_ = 600 nM) compared to the unmodified peptide (t_1/2_ = 2.1 h, 38 nM). In contrast, peptidomimetic **P2** maintained the potency of the parent compound (K_i_ = 37 nM) and increased the plasmatic half-life by 100% (t_1/2_ = 4.0 h). Nevertheless, these two peptide analogues were not further pursued as other strategies such as the incorporation of d-amino acids, β-amino acids or modification at the N-terminus were found to be more effective.

### 4.2. Leu-Enkephalin

The Dory group reported the synthesis of triazole-containing peptidomimetics based on Leu-enkephalin (Leu-Enk), an endogenous ligand for the delta opioid receptor (DOPr) [76]. Due to the rapid degradation of this pentapeptide, the group investigated the use of metabolically stable 1,2,3-triazoles to obtain analogues with increased biological half-lives. They designed four derivatives **Enk1–4** by systematically replacing each amide bond with a triazole (see Table 2). For the synthesis of these peptidomimetics, the triazole-bearing dipeptide was prepared in solution via CuAAC using CuI and 2,6-lutidine and the building blocks were then further used in the solid-phase synthesis of the peptidomimetics (Figure 3A). The binding affinities of all analogues were determined in a competitive binding assay on GH3 cell membrane extracts (rat pituitary tumour cell lines) containing DOPr and using the selective DOPr agonist [^3^H]-deltorphin II as competitive ligand. Compound **Enk1** with a triazole at position Phe^4^-Leu^5^ showed the highest affinity with an K_i_ = 89 nM, although it was 15-fold lower than the endogenous Leu-enkephalin (K_i_ = 6.3 nM) [85]. Derivative **Enk2** with a triazole at position Gly^3^-Phe^4^ displayed a 60-fold lower affinity (K_i_ = 460 nM) compared to the reference compound and the other analogues lost their affinity for DOPr (K_i_ > 1000 nM). Unfortunately, experiments regarding their stability are not reported. This study shows that the 1,2,3-triazole is not in all cases a universal bioisostere for a *trans*-amide bond as their incorporation into the peptide can result in diminished or abolished biological activity.

### 4.3. Bombesin

The amide-to-triazole switch was reported on the minimal binding sequence of bombesin BBN(7-14), an agonistic ligand of the gastrin-releasing peptide receptor (GRPR), which is overexpressed in a variety of tumours (i.e., prostate, breast, lung and pancreatic cancer) [77]. Valverde et al. chose this model peptide as an example for a radiolabelled tumour-targeting vector to demonstrate the potential of this novel peptide stabilisation method. The sequence was functionalised with a PEG_4_ spacer and the universal chelator 1,4,7,10-tetraazacyclododecane-1,4,7,10-tetraacetic acid (DOTA) at the N-terminus which was necessary for the radiolabelling with lutetium-177, a clinically established radionuclide. In addition, the methionine residue in position 14 was changed to norleucine (Nle) to avoid side products arising from the oxidation of methionine during the radiolabelling step [86]. Afterwards, a triazole scan of [Nle^14^]BBN(7-14) was performed by creating a library of nine different triazolo-peptidomimetics **BBN2–10** (Table 3) in which every amide bond was substituted with a triazole one at a time. The CuAAC was performed on solid phase according to Figure 3B by reacting the resin-bound azido-peptide with the corresponding α-amino alkyne, [Cu(CH_3_CN)]PF_6_, tris[(1-benzyl-1*H*-1,2,3-triazol-4-yl)methyl]amine (TBTA) and *N*,*N*-diisopropylethylamine (DIPEA) to achieve the formation of the 1,4-substituted 1,2,3-triazole at the desired position. Seven out of the nine peptidomimetics showed improved in vitro serum stability ranging from t_1/2_ = 8 h for **BBN8** to t_1/2_ = >100 h for **BBN4** (in comparison to t_1/2_ = 5 h for the reference peptide **BBN1**) thus enhancing the stability up to 20-fold. The amide-to-triazole switch in the two analogues **BBN2** and **BBN10** preserved the serum stability (t_1/2_ = 5 h and 6 h, respectively). The lipophilicity of all compounds was evaluated by determining the logD_7.4_ value. This is an important characteristic for radiopharmaceuticals as it impacts pharmacokinetic properties such as the renal excretion of the drug. Substitution of an amide bond with a 1,2,3-triazole did not significantly alter the lipophilicity of the novel peptide analogues at pH 7.4. When tested for their biological activity, the cell uptake and the dissociation constant (K_D_) were evaluated using GRPR-overexpressing PC3 cells (human prostate cancer cells). The internalisation rate of **BBN2**, **5** and **7** were comparable to the parental compound (29.1%, 28.3% and 24.5%, respectively compared to 27.7% for the unmodified reference peptide **BBN1**). The single digit nanomolar affinity of the reference compound **BBN1** to GRPR (K_D_ = 2.0 nM) was also maintained in **BBN2, 5** and **7** with K_D_ values of 3.0, 3.1 and 5.9 nM, respectively. For compound **BBN6**, the amide-to-triazole switch was slightly detrimental resulting in an internalisation rate of only 8.4% and a K_D_ value of 48.6 nM. The other derivatives had abolished biological function on GRPR. Thus, compound **BBN5** with the triazole situated between Gly^11^ and His^12^ was chosen for in vivo evaluation as the most promising candidate with a 3.5-fold increase in in vitro serum stability and conserved biological activity. It displayed in vivo specificity toward GRPR and a 2-fold higher tumour uptake in athymic nude mice bearing PC3 xenografts due to its higher in vivo stability.

Valverde et al. also explored the synthesis of triazolo-peptidomimetics of [Nle^14^]BBN(7-14) with multiple triazoles in the backbone [87]. Therefore, triazoles were strategically placed in the most promising positions as identified by the previous triazole scan. The three possible analogues **BBN11–13** with two triazoles in one molecule at positions Ala^9^-Val^10^, Val^10^-Gly^11^ or Gly^11^-His^12^ were synthesised as well as the peptidomimetic **BBN14** containing all three substitutions. Even though the half-lives in blood serum were increased 5- to 13-fold (27–66 h) compared to unmodified compound **BBN1**, the biological activity was lost (**BBN12–14**) or significantly decreased likely due to a constraint conformation of the peptidomimetic (K_D_ = 25.6 nM for **BBN11**, Table 3).

**Figure 5:**
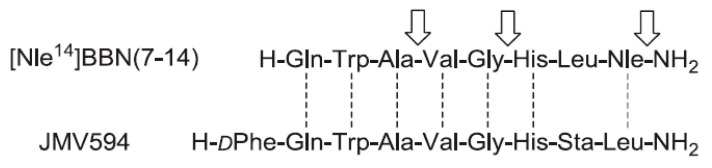
Comparison of the amino acid sequences of bombesin derivative [Nle^14^]BBN(7-14) and antagonist JMV594. Dashed lines illustrate the structural similarity of the amino acid sequences, and arrows indicate positions where amide bonds can be replaced by 1,2,3-triazoles in the agonist [Nle^14^]BBN(7–14) without loss of the biological properties of the vector. Reproduced with permission from Valverde et al. [89].

Another bombesin derivative studied by Valverde et al. was the antagonist JMV594 which differs from BBN(7-14) only in the last two amino acids at the C-terminus and the addition of a d-Phe at the N-terminus (Figure 5) [88,89]. 

Due to the high homology of the two peptides, the three most promising positions identified in the screening of [Nle^14^]BBN(7-14) were substituted with a triazole in JMV594. The obtained peptides were conjugated to PEG_4_ and DOTA for lutetium-177 chelation at their N-terminus and the resulting derivatives **BBN15–18** were tested for cell binding and receptor affinities (Table 4). The analogues **BBN17** and **BBN18** lost their biological activity and **BBN16** with the triazole at the C-terminus displayed a lower affinity (K_D_ = 8.1 nM) and cell binding (13%) compared to the reference compound **BBN15** (K_D_ = 2.7 nM, cell binding 30%). Surprisingly, in vitro blood serum stability tests showed that analogue **BBN16** as well as the unmodified peptide **BBN15**, have remarkably long half-lives in blood serum compared to agonistic [Nle^14^]BBN(7-14) (see above). 65% and 75% of the peptide and peptidomimetic were still intact after 48 h, respectively. Despite the high homology between the two bombesin derivatives, the successful results from the [Nle^14^]BBN(7-14) triazole screening could not be transferred to JMV594 analogues. These results underline the different mode of action of the two GRPR-binding molecules (agonistic vs. antagonistic peptides). In general, backbone modifications that are tolerated by one peptide cannot be applied automatically to a structurally related peptide.

### 4.4. Kisspeptin

Kisspeptin is an endogenous peptide involved in the modulation of the gonadotropic axis, a hormonal system including luteinising hormone (LH) and follicle-stimulating hormone (FSH) which regulates fertility in mammals and human. The downregulation of this system is the cause of severe reproductive disorders resulting in partial or complete infertility in humans [90,91]. It also occurs naturally in seasonal breeders such as sheep during the anoestrous phase and its manipulation can lead to a prolonged breeding season and improved livestock management [92]. It has been shown that injection of the truncated decapeptide analogue KP10 leads to an upregulation of the gonadotropic axis although the effect is only short-lasting due to quick degradation of the peptide by proteases. Beltramo et al. examined the stabilisation of the decapeptide **KP1**, the ewe analogue of KP10, via an amide-to-triazole switch [78]. First, they *N*-acetylated the endogenous decapeptide **KP1** which led to a highly improved resistance towards proteolysis as a small fraction (2.6%) of peptide was still present after 6 h of incubation in blood serum (**KP2**, Table 5). The amount of the reference compound **KP1** was below the detection limit at this time point. The potency of the novel compounds was determined in a calcium mobilisation assay using HEK-293 cells (human embryonic kidney cells) transfected with the kisspeptin receptor KISS1R. It was shown that *N*-acetylation of **KP1** also increased drug potency from an EC_50_ of 2.5 nM to 0.07 nM (**KP2**). In a next step, the two main proteolysis sites at position Phe^6^-Gly^7^ and Gly^7^-Leu^8^ were replaced by triazoles leading to two monotriazolo analogues **KP3** and **KP4** as well as bistriazolo-peptidomimetic **KP5**. The formation of these triazoles was accomplished on solid phase by the addition of Fmoc-protected α-amino alkynes, CuBr∙Me_2_S and DIPEA to the immobilised azidopeptide (Figure 3B). 

**Figure 6:**
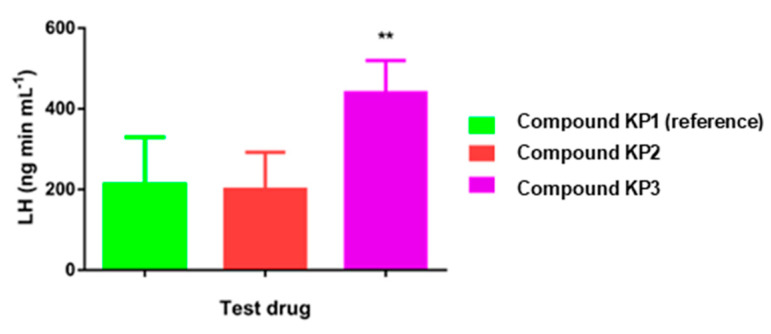
Total amount of luteinising hormone (LH) secreted in vivo in ewes after application of 5 nmol **KP1** (reference), **KP2** or **KP3**. Values are the mean ± SEM. ** for *p* < 0.01. Reproduced with permission from Beltramo et al. [78].

The in vitro serum stabilities of triazole-containing analogues **KP3–5** increased 15- to 25-fold compared to **KP2** as after 6 h of incubation 40.8, 61.4 and 50.7% of the peptidomimetics were still intact, respectively (compared to 2.6% of **KP2**). Regarding biological activity, **KP3** with the additional triazole at position Gly^7^-Leu^8^ maintained the potency of **KP2**. However, the amide-to-triazole switch in **KP4** was less favourable yielding in an EC_50_ of 0.6 nM and multiple triazole substitution in **KP5** was detrimental to the potency (EC_50_ = 120 nM). Compounds **KP2** and **KP3** were evaluated in vivo for their capacity to increase the plasma concentration of LH and FSH in anoestrous ewes. After intravenous injection, **KP2** did not change the LH concentration compared to **KP1** but compound **KP3** resulted in a clear increase (peak concentration: 5.4 ng mL^−1^ compared to 3.3 ng mL^−1^ for **KP1**). The duration of LH release after administration of **KP3** was also doubled in comparison to **KP1**, which in turn resulted in a higher amount of the hormone secreted over time (Figure 6). In vivo dose-response studies of **KP3** illustrated solely a modest increase in the maximal concentration, the duration of action and the total amount of LH secretion upon application of higher **KP3** concentrations. Similar trends were observed for secretion of FSH. Overall, these results were less significant than anticipated from the in vitro data probably due to fast renal excretion. These modifications, in combination with further optimisations, hold the potential to improve the treatment of reduced or complete infertility associated with the gonadotropic axis.

### 4.5. Neurotensin

**Figure 7:**
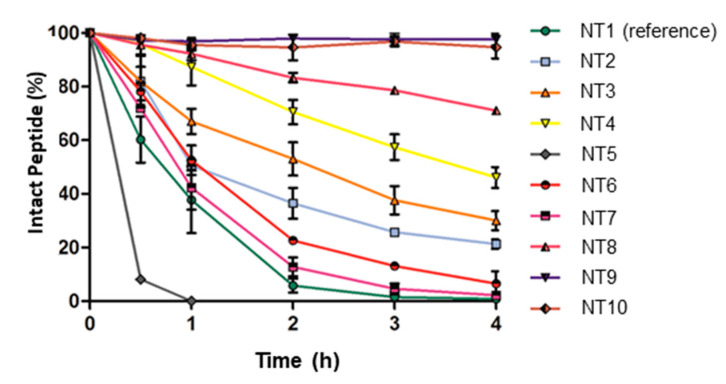
In vitro blood serum stabilities of reference compound **NT1** (green) and monotriazolo-peptidomimetics **NT2–10** at 37 °C. Values are expressed as percentage of intact peptide. Error bars indicate the standard deviation of mean values (n ≥ 2–3). Reproduced with permission from Mascarin et al. [93].

Mascarin et al. applied the amide-to-triazole switch approach to the stabilisation of the binding sequence of neurotensin, NT(8-13), a regulatory peptide which specifically binds to neurotensin receptors (NTR) [93]. The overexpression of subtype NTR1 in breast, pancreas, prostate cancer and many more makes this molecule an interesting candidate as a tumour targeting vector for the development of new therapeutic and diagnostic agents [94,95]. However, the major drawback is its low in vivo stability with a half-life of only a few minutes. Hence, the group was investigating the stabilisation of this peptide via a triazole scan. The binding sequence NT(8-13) was conjugated N-terminally to a PEG_4_ spacer and DOTA, which was employed for the radiolabelling with lutetium-177. A first generation of triazolo-peptidomimetics was obtained by the stepwise substitution of each amide bond with a 1,4-disubstituted 1,2,3-triazole except for the amide bond between Arg^9^-Pro^10^, which does not allow for an amide-to-triazole switch due to the secondary amine of Pro. A second generation was obtained by replacing isoleucine in position 12 of the most promising triazolo-peptidomimetics with *tert*-leucine (Tle), another strategy reported for the stabilisation of this sequence. The same synthetic protocol as described above for bombesin analogues was employed (Figure 3B) [77]. All compounds were then evaluated in vitro for their stability in blood serum (Figure 7) as well as their cell internalisation into NTR1-expressing HT29 cells (human colon carcinoma) and affinity toward the NTR1 (Table 6). Substitution of amide bonds between the C-terminus and Pro^10^ (compounds **NT2–5**) resulted in a loss of biological function albeit an up to 4-fold increase in vitro stability was achieved (t_1/2_ = 13.0–164.0 min compared to t_1/2_ = 39.4 min for reference compound **NT1**). This came as no surprise, as this region is involved in several H-bond interactions with the receptor and therefore sensitive to structural modifications. Exchanging the amide bond at the N-terminus PEG_4_-Arg^8^ or Arg^8^-Arg^9^ (compound **NT6–7**) slightly improved in vitro stability to half-lives of 64.9 min and 46.9 min, respectively. Biological evaluation of **NT6** and **NT7** showed that the activity was maintained in vitro with a cellular uptake of 6.4% and 9.4%, respectively, and K_D_ values of 8.8 nM and 4.5 nM (for comparison, reference compound **NT1**: 7.3% internalisation and K_D_ 3.7 nM). Earlier studies reported the remarkably improved stability of NT(8–13) when Ile^12^ is replaced with a Tle residue [96,97]. Modification of the two most promising triazolo-peptidomimetics **NT6** and **NT7** with the Ile^12^-to-Tle^12^ switch resulted in derivatives **NT9** and **NT10** with a 100-fold increase of the in vitro stability (>95% of compound still intact after 4 h; half-lives not determined) compared to the reference compound **NT8** (0.9% after 4 h). However, only derivative **NT9** retained a submicromolar affinity toward NTR1 (K_D_ = 214 nM). In vivo evaluation of the most promising peptide conjugates in athymic nude mice bearing HT-29 xenografts revealed that the replacement of the Arg^8^-Arg^9^ amide bond by a triazole resulted in two-fold tumour uptake for both, the Ile^12^ derivative **NT6** and the Tle^12^ derivative **NT9**, compared to their respective reference compounds without a triazole. This can be ascribed to the significantly improved stability of the triazolo-peptidomimetics. Compound **NT7** on the other hand, showed a high tumour-to-background ratio, a property which is particularly important for potential imaging applications.

Subsequent investigations focused on the incorporation of multiple triazoles in the backbone of **NT(8–13)** [98]. Building on the results of the triazole scan discussed above, the triazolo-peptidomimetics **NT11** and **NT12** with a simultaneous substitution at PEG_4_-Arg^8^ and Arg^8^-Arg^9^ for the Ile^12^ and Tle^12^ analogue were synthesised and tested in vitro. Multiple triazoles could not improve the stability of the peptidomimetics further. Surprisingly, the biological activity of **NT11** was maintained (k_D_ = 4.6 nM, 10.8% cell internalisation) but unexpectedly the stability was found to be reduced (t_1/2_ = 13 min) compared to the unmodified peptide conjugate **NT1** (t_1/2_ = 39.4 min). One explanation might be that the introduction of two consecutive triazoles in this peptide sequence results in a confirmation of the peptide, which is prone to enzymatic degradation. In the case of the Tle^12^ derivative **NT12**, the in vitro plasma stability was maintained (>97% intact after 4 h), but the receptor affinity was abolished.

### 4.6. Cathepsin K & S Inhibitors

The Lalmanach group explored the stabilisation of peptidic inhibitors for cathepsin K and S [99]. These two proteins are cysteine proteases normally present in lysosomes and share a high homology. When found in the extracellular matrix, these enzymes are often overexpressed and dysregulated, making them innovative targets for new therapies for a range of medical conditions. Cathepsin K is considered as a target involved in osteoporosis whereas cathepsin S has been identified to play a role in autoimmune diseases and neuropathic pain [100,101]. One strategy to identify inhibitors for proteases is to turn their substrates into a non-cleavable inhibitory molecule by substituting the hydrolysed amide bond. For this purpose, the use of metabolically stable 1,2,3-triazoles as amide bond mimics at the previously identified scissile bonds in two known cathepsin K and S inhibitors were investigated. They also compared the resulting triazolo-peptidomimetics to their azapeptide counterparts in which the α-carbon next to the labile amide bond was replaced by a nitrogen (Figure 1). The triazolo-peptidomimetics were synthesised on solid phase following the protocol of Beltramo et al. (Figure 3B) [78]. The substrates were tested for their ability to inhibit enzyme activity on a range of different proteases (including human cysteine proteases, a matrix metalloproteinase, an aspartyl protease and several serine-proteases) and K_i_ values for cathepsin K and S were determined using fluorescently-labelled substrates (Table 7). Triazole-containing compound **CatS1** was based on the previously reported sequence of a tridecapeptide. [102] It was shown that it inhibits cathepsin S, but also cathepsin K and L without affecting any other protease. This peptidomimetic was not cleaved by cathepsin S, K or L as shown by HPLC analyses of the different incubation mixtures. It was further demonstrated that **CatS1** reversibly and competitively inhibits cathepsins S, K and L in the micromolar range (K_i_ = 15, 10 and 30 μM, respectively). In contrast to other cathepsins present in the lysosome, cathepsin S is not only operating at acidic pH but also remains stable and active at neutral pH. Indeed, K_i_ values obtained at pH 5.5 and pH 7.4 were comparable for cathepsin S (K_i_ = 15 vs. 42 μM) but not for cathepsin K or L (data not shown here). However, the corresponding azapeptide **CatS2** was the more potent and specific inhibitor of cathepsin S and K with a K_i_ value in the nanomolar range.

Another set of investigated peptidomimetics was based on a bradykinin-derived substrate, previously reported to be degraded by cathepsin K [103]. Triazolo-peptidomimetic **CatK1** did not inhibit cathepsin S and other proteases but was able to inhibit cathepsin K and L in a reversible manner with a K_i_ of 0.8 and 22 μM, respectively. However, azapeptide **CatK2** displayed a much higher affinity in the nanomolar range. Molecular modelling studies suggest that although the triazole exhibits similar geometric, steric and electronic features as the *trans*-amide bond, its constrained coplanar structure reduces the interaction of the peptidomimetics with the enzyme’s active pocket. Hence, in this case the insertion of a 1,2,3-triazole turned out to be less effective than the substitution of amide bonds with a semicarbazide due to significantly lower affinities (100- to 1000-fold).

### 4.7. Caspase-3 Inhibitors

**Figure 8:**
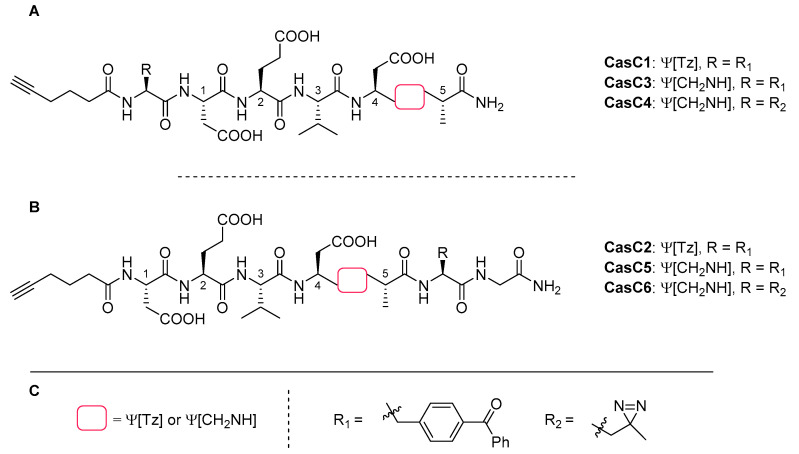
**A:** Structures of **CasC1, CasC3, CasC4** with the photo-activable crosslinker R at the N-terminus; **B**: structures of **CasC2, CasC5, CasC6** with the photo-activable crosslinker R at the C-terminus; **C**: Structures of backbone modification (red box, either Ψ[Tz] or Ψ[CH_2_NH]) at Asp^4^-Ala^5^ and of the photo-cleavable linkers.

Proteases are ubiquitous enzymes executing various tasks in organisms and their activity is tightly regulated by different mechanisms. A lot of effort has been invested in studying their function and localisation. For example, Verhelst and co-workers investigated the possibility to develop a general strategy for the design of chemical probes that covalently interact with proteases [104]. Their idea was to design inhibitors based on known substrates by substituting the labile peptide bond with a metabolically stable surrogate and attach a photo-crosslinker to covalently inhibit the targeted enzyme. They studied caspase-3 as a model protease and developed several peptidomimetics based on the known pentapeptide substrate Asp-Glu-Val-Asp-Ala (Figure 8). An alkyne function was localised as a bioorthogonal tag at the N-terminus of the peptide to enable the installation of a fluorophore via CuAAC. In addition, a glycine residue with a benzophenone moiety at the α-carbon serving as photoactivatable crosslinker was incorporated at either the C-terminus (**CasC1, CasC3, CasC4**) or the N-terminus (**CasC2, CasC5, CasC6**) of the peptide. The two peptide analogues **CasC1** and **CasC2** were obtained by an amide-to-triazole switch at the previously identified scissile bond Asp^4^-Ala^5^ of the parent peptide. The other probes **CasC3–6** contained reduced amide bonds in the same position and either benzophenone or diazirine as substituent of the glycine residue at the C- or N-terminus (Figure 8).

The synthesis of the triazolo-peptidomimetics was performed on solid phase, including the triazole incorporation via CuAAC (Figure 3B). For this step, immobilised azidopeptide was incubated with the corresponding protected α-amino alkyne, CuBr, Na-ascorbate, 2,6-lutidine and DIPEA. With the chemical probes in hand, they first performed competitive activity-based protein profiling (ABPP) to investigate the inhibitory characteristics of the novel compounds. For this purpose, caspase-3 was incubated with the probes under UV irradiation and residual caspase-3 activity was measured using a fluorescent enzyme-specific probe and analysed by SDS-PAGE. Analogues **CasC3–6** with a reduced amide bond at Asp^4^-Ala^5^ resulted in complete inhibition of the protease whereas triazole-containing derivative **CasC1** did not show any inhibitory effect. The second triazolo-peptidomimetic **CasC2** displayed slight inhibition at 10 μM but even increasing the substrate concentration to 100 μM did not lead to full inhibition of caspase-3. Similar to the findings for cathepsin K & S inhibitors described above, triazoles were the less efficient amide bond surrogate for caspase-3 inhibitors probably due to the increased rigidity of the triazole compared to the amide bond. Further studies are necessary to determine if this is also valid for other proteases inhibitors.

### 4.8. NRP-1/VEGF_165_ Inhibitors

Neuropilin-1 (NRP-1) is a protein implicated in angiogenesis and therefore also plays a role in the vascularisation of tumours [105]. It has been shown that it is heavily overexpressed in a variety of tumours and hence, related to tumour malignancy and poor prognosis [106,107]. The interaction of NRP-1 with vascular endothelial growth factor 165 (VEGF_165_) is an interesting target for novel cancer therapies and several inhibitors of NRP-1/VEGF_165_ have been proposed as potential drugs such as the heptapeptide **A7R**. [108] Based on these findings, the group of Misicka has developed branched pentapeptides of the type Lys(Har)-Xaa-Xaa-Arg (Har = homoarginine) with submicromolar IC_50_ values, for example 0.3 μM for Xaa-Xaa= Pro-Ala (**NV1**). However, they observed enzymatic cleavage of the peptides, which led to a low blood plasma stability in vitro [109]. To overcome the peptide’s instability, the authors turned their attention to 1,4-disubstituted 1,2,3-triazoles as a stable amide bond surrogate. They designed and synthesised a series of peptides where they connected the C-terminal Arg residue and the N-terminal branched Lys(Har) residue (either l-Lys or d-Lys) with different peptidomimetic spacers. The spacers consisted of a short sequence of one to three amino acids in which at least one amide bond was substituted with a triazole (Table 8) [79]. In total, seven spacers varying in length and the number of triazole bonds were investigated (not all of them are shown here). Synthesis of the triazolo-peptidomimetics was achieved on solid support (Figure 3B) with CuSO_4_·5H_2_O and sodium ascorbate as catalysts in the CuAAC. The inhibitory activity of all derivatives towards binding of VEGF_165_ to NRP-1 was measured at a concentration of 10 μM using an enzyme-linked immunosorbent assay (ELISA) and IC_50_ values were determined for the most active compounds. None of the triazolo-peptidomimetics described performed better than reference **A7R**. Two compounds **NV2** and **NV3** of the series displayed inhibition comparable to A7R (58% and 52% inhibition at 10 μM, respectively, compared to 61% for A7R). Both peptidomimetics contain the GlyΨ[Tz]GlyΨ[Tz] motif as a linker and differ in the chirality of the terminal Lys residue (l vs. d, respectively). However, the in vitro plasma stability results revealed that these two analogues are significantly more stable. After 48 h, 70% of compound **NV2** and >90% of compound **NV3** were still intact (half-lives not determined), whereas the previously reported compound **NV1** possess a half-life of only 39 min [109]. Further modification of **NV2** at the N-terminus was attempted to optimise the inhibition of NRP-1/VEGF_165_ interaction but efforts remained unsuccessful. Molecular dynamics simulations of compound **NV2** bound to NRP-1 confirmed a relatively instable binding mode, which is in line with lower inhibitory activity compared to **A7R**. The simulations revealed that even when the C-terminus is tightly bound to the receptor, the triazole-containing spacer and the N-terminus still showed significant mobility which prevents stable inhibitor-protein interaction. This suggests that improved inhibition could be achieved by introducing more rigid structures and/or groups that enable further interaction with the receptor. In conclusion, **NV2** and **NV3** demonstrated a remarkably improved in vitro stability due to the triazole insertion in the peptide’s backbone although no enhanced inhibitory activity compared to the reference compound **A7R** or **NV1** was observed. These results indicate that the compounds **NV2** and **NV3** could serve as lead structures in the development of more active NRP-1/VEGF_165_ inhibitors.

### 4.9. Minigastrins

**Figure 9:**
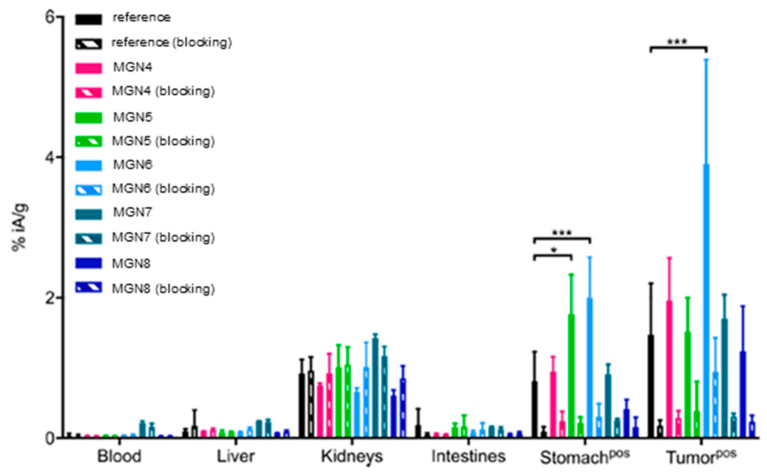
Biodistribution of ^177^Lu-labelled reference compound and **MG4–8** in mice bearing A431-CCK2R tumour xenografts 4 h post-injection of the radiotracer. Percentage of injected activity per gram of organ/tissue (%iA/g) without (plain columns) and with (crosshatched columns) co-injection of excess minigastrin (blocking experiments to verify receptor specificity). * for *p* < 0.033 and *** for *p* < 0.001. Reproduced with permission from Grob et al. [82].

Minigastrin is a tridecapeptide binding to the cholecystokinin-2-receptor (CCK2R), a receptor whose overexpression is linked to various cancer types such as pancreatic, thyroid or neuroendocrine tumours [110]. Minigastrin analogues have been extensively explored as potential tumour-targeting vectors for CCK2R-positive cancer cells and a screening test resulted in the truncated DOTA-conjugated octapeptide MG11 as a promising candidate with a high receptor affinity but poor plasma stability [111]. Recently, Grob et al. reported the successful application of the amide-to-triazole switch methodology to MG11 in order to increase its stability [82]. The authors focussed on the use of MG11 as a radiotracer. For easier handling, they replaced the methionine residue in position 15 with norleucine **[Nle^15^]MG11**. [86] They then performed a triazole scan, where every peptide bond was substituted individually with a 1,4-disubstituted-1,2,3-triazole. Synthesis of these triazolo-peptidomimetics **MGN1–8** was performed on solid phase according to procedures described by Valverde et al. (Figure 3B) [77]. In vitro blood plasma stability tests revealed that six out of eight compounds showed enhanced stability and in five cases even more than 50% of the molecules were still being completely intact after 24 h of incubation (Table 9). It was calculated that the half-life of compound **MGN4** with a triazole at position Trp^14^-Nle^15^ is 90-fold higher (t_1/2_ = 349.8 h) compared to the reference compound **[Nle^15^]MG11** (t_1/2_ = 3.9 h). Insertion of a triazole at position Gly^13^-Trp^14^ (**MGN5**) displayed maintained stability (t_1/2_ = 3.8 h) and only substitution at Tyr^12^-Gly^13^ (**MGN6**) resulted in a slight decrease of the proteolytic stability of the peptide (t_1/2_ = 2.6 h). The measurement of logD_7.4_ values of all compounds confirmed earlier findings of the same group, that the lipophilicity was not significantly altered by the introduction of a triazole into the peptide backbone. [77] All compounds were evaluated in vitro for their internalisation into CCK2R-expressing A431 cells (human epidermoid carcinoma) and affinity toward CCK2R (Table 9). It was shown that compounds **MGN1–3** with a triazole incorporated within the last three amino acids of the C-terminus almost completely abolished their biological activity. This comes as no surprise as this region is the minimal binding sequence and known to be very sensitive even to minor modifications. Compounds **MGN4**, **MGN5** and **MGN7** maintained their biological activity (i.e., 33.1% of internalisation after 4 h and IC_50_ = 25.4 nM for **MGN4**) when compared to reference compound **[Nle^15^]MG11** (32.2% internalisation, IC_50_ = 15.4 nM). **MGN6** and **MGN8** were reported to have increased biological activity. Interestingly, compound **MGN6** which displayed the lowest in vitro serum stability almost doubled its internalisation rate to 54.3% and enhanced the IC_50_ value to 1.7 nM. This is the first example of an amide-to-triazole switch leading to higher receptor affinity of the resulting peptidomimetic. Compounds with maintained or improved biological activity were also tested in vivo in athymic nude mice with CCK2R-positive tumour xenografts (Figure 9). Not surprisingly, compound **MGN6** also performed best in mice with a 2.6-fold increased tumour uptake and a slower tumour washout. In addition, the analogue also showed a 3-fold increased tumour-to-kidney ratio, a property important for the translation of radiotracers to the clinic as the dose to radiation-sensitive kidneys needs to be minimised. Despite its slightly reduced in vitro plasma stability, derivative **MGN6** performed better than other minigastrin derivatives because of its increased affinity and tumour uptake. Further in silico modelling studies led to the hypothesis that an additional cation-π interaction between the 1,2,3-triazole in **MGN6** and Arg^356^ in the binding pocket of the receptor are responsible for the improved binding properties of compound **MGN6** to the receptor. Thus, this is the first example of the application of an amide-to-triazole switch which resulted in a derivative with improved pharmacodynamic properties such as receptor affinity and cell internalisation.

Based on these results, Grob et al. studied the incorporation of multiple triazoles in the peptide backbone of **[Nle^15^]MG11** for potential additive or synergistic effects in order to further improve the properties of the peptide [112]. Therefore, the authors were strategically combining positions for amide-to-triazole switches, which led to increased stability or affinity in the case of **[Nle^15^]MG11** (Table 9). In total, seven novel multi-triazole containing peptide analogues **MGN9–15** were synthesised and tested for their in vitro and in vivo properties. Except for **MGN10**, they all displayed maintained or improved half-lives in blood serum compared to **[Nle^15^]MG11** ranging from 4.1 to 386.1 h. In five out of seven cases, the in vitro stability fell in between or was above the half-lives of the corresponding monotriazolo-peptidomimetics. Incorporation of multiple triazoles into the backbone of **[Nle^15^]MG11** was slightly detrimental to cell internalisation for **MGN9** and **MGN11** (28.2% and 31.1%, respectively), but beneficial for all other tested compounds, with **MGN13** having the highest internalisation rate of 58.4%. A similar trend was observed for the affinities, where all compounds except **MGN9** and **MGN11** displayed maintained or improved IC_50_ values, ranging from 5.3 to 15.6 nM. All examples except **MGN11** were included in in vivo studies and displayed higher tumour uptake than the reference compound **[Nle^15^]MG11**. The most promising candidates were **MGN13** and **MGN15** containing further triazole substitutions at position D-Glu^10^-Ala^11^ and/or Ala^11^-Tyr^12^ in addition to the modification at Tyr^12^-Gly^13^. In vivo, the multitriazolo-peptidomimetics showed a further 1.5-fold increase in tumour uptake and a 2- to 3-fold increase in the tumour-to-kidney ratio in athymic nude mice with CCK2R-positive tumour xenografts compared to mono-triazole compound **MGN6**. These results confirm that the amide-to-triazole switch is a promising strategy to obtain peptidomimetics with improved tumour-targeting properties. In summary, **[Nle^15^]MG11** is highly tolerant towards backbone modifications in its N-terminal region whilst preserving or improving receptor affinity. This is the first example of multiple triazole-to-amide switches in peptides leading to improved serum stability and affinity to the receptor. In particular, **MGN15** is the first reported compound where three amide bonds in the backbone were substituted with a triazole and biological function was still maintained. However, no apparent trend for synergistic or additive effects of multiple triazoles in the backbone of peptides can currently be defined to allow the prediction of the biological behaviour of multitriazolo-petidomimetics based on those of the corresponding monosubstituted triazolo-peptidomimetics.

### 4.10. Angiotensin

Angiotensin II (**AII**) is an octapeptide and serves as ligand for the angiotensin receptor subtype 1 and 2 (AT1R and AT2R). It has been shown that AT2R represents a novel target for the development of treatments for various cancers including melanoma and breast cancer [113,114]. Nevertheless, there is still an imminent need to develop ligands with high receptor subtype-specificity (Table 10). One potent and selective derivative of **AII** is the previously reported **[Tyr^6^]AII**, in which His^6^ is replaced by Tyr [114]. This modification yielded in a ligand with nanomolar affinity for AT2R and an 18.000-fold selectivity for AT2R over AT1R. However, its use is limited by a low metabolic stability. Therefore, Tzakos and co-workers intended to develop more stable analogues by applying the amide-to-triazole switch to **[Tyr^6^]AII**. [115] They introduced a 1,2,3-triazole at four strategic positions in the peptide backbone yielding triazolo-peptidomimetics **AII1–4** (Table 10). The triazoles were incorporated following the protocol from Valverde et al. (Figure 3B) [77].

**Figure 10:**
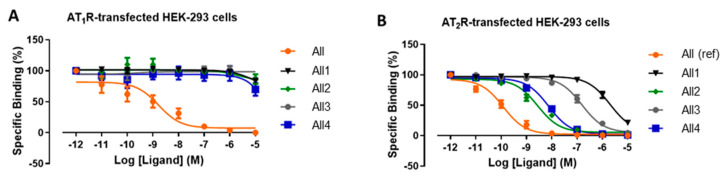
Competition-binding experiment for AII (reference compound) and the four peptidomimetic analogues **All1–4** against [^125^I,Sar^1^,Ile^8^]AII on AT1R-transfected HEK-293 cells (**A**) and AT2R-transfected HEK-293 cells (**B**). Reproduced with permission from Vrettos et al. [115].

These novel triazolo-peptidomimetics were tested for their plasma stability. After 24 h, only approx. 10% of the compounds were found degraded, compared to 46% of the reference compound [Tyr^6^]AII. Thus, the replacement of amide bonds in the backbone of the peptide of investigation significantly enhanced its stability in blood plasma. The binding affinity and selectivity of the peptidomimetics and reference compound **AII** was assessed in a competitive binding assays using HEK-293 cells (human embryonic kidney cells) transfected with AT1R or AT2R and [^125^I,Sar^1^,Ile^8^]AII as competitive ligand (Figure 10). The affinities of all compounds were reduced when compared to the endogenous ligand **AII**. Interestingly, the experiments revealed that two of the novel derivatives retained high selectivity for AT2R over AT1R (3.600- and 1.300-fold for compound **AIl2** and **AII4**, respectively) whereas the two other peptidomimetics only showed minimal selectivity (64- and 5-fold for compound **AII3** and **AII1**, respectively). It should be noted that the AT2R selectivities represent conservative estimations as the analogues failed to displace AT1R binding sufficiently. Further high-resolution 2D NMR studies confirmed that compounds with maintained receptor subtype selectivity, **AII2** and **AII4**, shared structural features in solution which indicates similar conformation. They clearly clustered differently from compounds **AII1** and **AII3** which exhibit poor receptor subtype selectively. This could imply that the introduction of a triazole moiety at specific positions favours a structural microenvironment that is responsible for the observed higher affinities of **All2** and **All4** to AT2R. Thus, these results illustrate that the amide-to-triazole switch can also provide peptide analogues of enhanced metabolic stability and maintained receptor subtype selectivity.

## 5. Conclusions

In this review we discussed the use of 1,4-disubstituted 1,2,3-triazoles as bioisosteres for a *trans*-amide bond in peptidomimetics in order to enhance the parent peptide’s metabolic stability whilst retaining or improving its biological activity.

In the majority of the reported cases, the substitution of an amide bond with a 1,4-disubstituted 1,2,3-triazole led to peptidomimetics with increased in vitro blood serum stability. These results underline the importance and thus growing interest in triazoles as promising amide bond surrogates. However, a second important factor that needs to be considered is the impact of such structural modifications on the peptides’ biological activity, for example, the affinity or selectivity towards its respective receptor. Another important parameter in drug development is the lipophilicity of the compound. It has been shown that the amide-to-triazole switch leads to peptidomimetics with maintained lipophilicity. In the studied cases, single or multiple substitutions of (up to three) amide bonds with triazoles did not change the logD_7.4_ values of peptides consisting of at least six amino acids [77,82,93,112]. Even though 1,4-disubstituted 1,2,3-triazoles are good bioisosteres of *trans*-amide bonds by mimicking electronic and steric features, a few essential differences exist. First, the heterocycle slightly increases the distance between the two neighbouring amino acid residues when compared to a peptide bond. Triazoles also possess a higher rigidity and planarity which can result in changes of the conformation of the peptide that can potentially influence the binding to its biological target. Furthermore, the introduced heterocycle may enable new π-interactions between the peptidic ligand and the binding site of its respective receptor, which in turn can influence its biological properties. In general, the affinity and selectivity a novel triazolo-peptidomimetic towards their receptor do not seem to be predictable. Ideally a triazole-scan, similar to established Ala-scans, is performed with the peptide of interest to evaluate all positions within a peptide sequence in order to identify the most promising one(s) which tolerate(s) the structural modification. The amide-to-triazole switch can lead to a higher success rate when compared to other established stabilisation methods such as in the case of BBN(7-14), where four novel peptidomimetics with conserved biological activity were identified whereas the reduction of amide bonds or *N*-methylation recognised only two novel promising compounds each [44,116,117]. On the other hand, in the case of protease inhibitors for caspase-3 or cathepsin K & S, the amide-to-triazole switch was not successful and more promising results were obtained with reduced amide bonds (for caspase-3) or the corresponding azapeptides (for cathepsin K & S) [99,104]. In the same way, additive or synergistic effects of combining multiple triazoles in one peptide appear to be difficult to anticipate based on the data of mono-substituted triazolo-peptidomimetics. In addition, the combination of this stabilisation strategy with other backbone modifications such as reduced amide bonds or *N-*methylated amino acids are still outstanding and leaves ample possibilities to explore in the future. In conclusion, an amide-to-triazole switch is often in favour of higher in vitro blood serum stabilities, but the biological properties of the resulting peptidomimetics need to be evaluated individually in order to develop triazolo-peptidomimetics successfully as potential drug candidates.

## Figures and Tables

**Table 1:** Sequences of Multi-Leu and **P1–2** and their reported biological properties [84].

Compound	Sequence	t_1/2_ [h] ^b^	K_i_ [nM]
**Multi-Leu ^a^**	Ac-Leu-Leu-Leu-Leu-Arg-Val-Lys-Arg-NH_2_	2.1 ± 0.2	38
**P1**	Ac-Leu-Leu**Ψ[Tz]**Leu-Leu-Arg-Val-Lys-Arg-NH_2_	1.0 ± 0.2	600
**P2**	Ac-Leu**Ψ[Tz]**Leu-Leu-Leu-Arg-Val-Lys-Arg-NH_2_	4.0 ± 0.5	37

^a^ Reference compound. ^b^ Determined in blood plasma.

**Table 2:** Sequences of Leu-Enk and **Enk1–4** and their reported K_i_ values [76].

Compound	Sequence	K_i_ [nM] ^b^
**Leu-Enk ^a^**	Tyr-Gly-Gly-Phe-Leu	6.3 ± 0.9
**Enk1**	Try-Gly-Gly-Phe**Ψ[Tz]**Leu	89 ± 12
**Enk2**	Try-Gly-Gly**Ψ[Tz]**Phe-Leu	460 ± 250
**Enk3**	Try-Gly**Ψ[Tz]**Gly-Phe-Leu	>1000
**Enk4**	Try**Ψ[Tz]**Gly-Gly-Phe-Leu	>1000

^a^ Reference compound. ^b^ K_i_ values were determined in competitive binding assays on GH3/DOPr cell membrane extracts with [^3^H]-deltorphin II as selective agonist. K_i_ values are expressed as the means ± standard error of means (SEM) of three to four independent experiments.

**Table 3:** Sequences of **BBN1–14** and their reported biological properties [77,87].

Comp.	Sequence	t_1/2_ [h] ^b^	Uptake after 4 h [%] ^c^	K_D_ [nM] ^d^
**BBN1 ^a^**	[^177^Lu]Lu-DOTA-PEG_4_-Gln-Trp-Ala-Val-Gly-His-Leu-Nle-NH_2_	5	27.7	2.0 ± 0.6
**BBN2**	[^177^Lu]Lu-DOTA-PEG_4_-Gln-Trp-Ala-Val-Gly-His-Leu-Nle**Ψ[Tz]**H	6	29.1	3.0 ± 0.5
**BBN3**	[^177^Lu]Lu-DOTA-PEG_4_-Gln-Trp-Ala-Val-Gly-His-Leu**Ψ[Tz]**Nle-NH_2_	60	0.2	n.d.
**BBN4**	[^177^Lu]Lu-DOTA-PEG_4_-Gln-Trp-Ala-Val-Gly-His**Ψ[Tz]**Leu-Nle-NH_2_	>100	n.o.	n.d.
**BBN5**	[^177^Lu]Lu-DOTA-PEG_4_-Gln-Trp-Ala-Val-Gly**Ψ[Tz]**His-Leu-Nle-NH_2_	17	28.3	3.1 ± 1.0
**BBN6**	[^177^Lu]Lu-DOTA-PEG_4_-Gln-Trp-Ala-Val**Ψ[Tz]**Gly-His-Leu-Nle-NH_2_	25	8.4	48.6 ± 11.5
**BBN7**	[^177^Lu]Lu-DOTA-PEG_4_-Gln-Trp-Ala**Ψ[Tz]**Val-Gly-His-Leu-Nle-NH_2_	16	24.5	5.9 ± 1.8
**BBN8**	[^177^Lu]Lu-DOTA-PEG_4_-Gln-Trp**Ψ[Tz]**Ala-Val-Gly-His-Leu-Nle-NH_2_	8	n.o.	n.d.
**BBN9**	[^177^Lu]Lu-DOTA-PEG_4_-Gln**Ψ[Tz]**Trp-Ala-Val-Gly-His-Leu-Nle-NH_2_	14	n.o.	n.d.
**BBN10**	[^177^Lu]Lu-DOTA-PEG_4_**Ψ[Tz]**Gln-Trp-Ala-Val-Gly-His-Leu-Nle-NH_2_	5	0.5	n.d.
**BBN11**	[^177^Lu]Lu-DOTA-PEG_4_-Gln-Trp-Ala**Ψ[Tz]**Val-Gly**Ψ[Tz]**His-Leu-Nle-NH_2_	27	21.7 ± 0.2	25.6 ± 6.9
**BBN12**	[^177^Lu]Lu-DOTA-PEG_4_-Gln-Trp-Ala-Val**Ψ[Tz]**Gly**Ψ[Tz]**His-Leu-Nle-NH_2_	40	3.5 ± 0.6	>1000
**BBN13**	[^177^Lu]Lu-DOTA-PEG_4_-Gln-Trp-Ala**Ψ[Tz]**Val**Ψ[Tz]**Gly-His-Leu-Nle-NH_2_	66	0.1 ± 0.1	>1000
**BBN14**	[^177^Lu]Lu-DOTA-PEG_4_-Gln-Trp-Ala**Ψ[Tz]**Val**Ψ[Tz]**Gly**Ψ[Tz]**His-Leu-Nle-NH_2_	61	0.3 ± 0.1	>1000

^a^ Reference compound. ^b^ Determined in blood serum at 37 °C. ^c^ Ratio of specific receptor-bound and cell-internalised compound expressed in % of administered dose. Expressed as the means ± SEM of at least two independent experiments. n.o.: not observed ^d^ Determined by receptor saturation binding assay on PC3 cells expressing GRPr. Expressed as the means ± SEM of at least two independent experiments. n.d.: not determined.

**Table 4:** Sequences of **BBN15–18** and their reported biological properties [89].

Comp.	Sequence ^b^	Stability after 48 h [%] ^c,d^	Uptake after 4 h [%] ^d,e^	K_D_ [nM] ^f^
**BBN15 ^a^**	[^177^Lu]Lu-DOTA-PEG_4_-d-Phe-Gln-Trp-Ala-Val-Gly-His-Sta-Leu	65	30	2.7
**BBN16**	[^177^Lu]Lu-DOTA-PEG_4_-d-Phe-Gln-Trp-Ala-Val-Gly-His-Sta-Leu**Ψ[Tz]**H	75	13	8.1
**BBN17**	[^177^Lu]Lu-DOTA-PEG_4_-d-Phe-Gln-Trp-Ala-Val-Gly**Ψ[Tz]**His-Sta-Leu	n.d.	n.d.	>1000
**BBN18**	[^177^Lu]Lu-DOTA-PEG_4_-d-Phe-Gln-Trp-Ala**Ψ[Tz]**Val-Gly-His-Sta-Leu	n.d.	n.d.	>1000

^a^ Reference compound. ^b^ Sta: statine: (3*S*,4*S*)-4-amino-3-hydroxy-6-methylheptanoic acid. ^c^ Determined in blood serum at 37 °C. Expressed as % of intact peptide. ^d^ n.d.: not determined ^e^ Ratio of specific receptor-bound and cell-internalised compound expressed in % of administered dose. Expressed as the means of three independent experiments. ^f^ Determined by receptor saturation binding assay on PC3 cells expressing GRPr. Expressed as the means ± SEM of at least two independent experiments.

**Table 5:** Sequences of **KP1–5** and their reported biological properties [78].

Comp.	Sequence	Stability after 6 h [%] ^b,c^	EC_50_ [nM] ^d^
**KP1 ^a^**	Tyr-Asn-Trp-Asn-Ser-Phe-Gly-Leu-Arg-Tyr-NH_2_	n.d.	2.5 ± 2.2
**KP2**	Ac-Tyr-Asn-Trp-Asn-Ser-Phe-Gly-Leu-Arg-Tyr-NH_2_	2.6 ± 0.4	0.07 ± 0.06
**KP3**	Ac-Tyr-Asn-Trp-Asn-Ser-Phe-Gly**Ψ[Tz]**Leu-Arg-Tyr-NH_2_	40.8 ± 2.9	0.07 ± 0.06
**KP4**	Ac-Tyr-Asn-Trp-Asn-Ser-Phe**Ψ[Tz]**Gly-Leu-Arg-Tyr-NH_2_	61.4 ± 6.4	0.6 ± 0.05
**KP5**	Ac-Tyr-Asn-Trp-Asn-Ser-Phe**Ψ[Tz]**Gly**Ψ[Tz]**Leu-Arg-Tyr-NH_2_	50.7 ± 3.2	120 ± 87

^a^ Reference compound. ^b^ Determined in blood serum at 39 °C. ^c^ n.d.: not detectable ^d^ Determined by a calcium mobilization assay on HEK-293 cells transfected with KISS1R.

**Table 6:** Sequences of **NT1–12** and their reported biological properties [93,98].

Comp.	Sequence	Stability after 4 h [%] ^b^ (t_1/2_ in min) ^c^	Uptake after 4 h [%] ^d^	K_D_ [nM] ^e^
**NT1 ^a^**	[^177^Lu]Lu-DOTA-PEG_4_-Arg-Arg-Pro-Tyr-Ile-Leu	0.9 ± 0.3 (39.4)	7.3. ± 0.4	3.7 ± 0.8
**NT2**	[^177^Lu]Lu-DOTA-PEG_4_-Arg-Arg-Pro-Tyr-Ile-Leu**Ψ[Tz]**H	21.3 ± 1.8 (69.7)	n.o.	n.d.
**NT3**	[^177^Lu]Lu-DOTA-PEG_4_-Arg-Arg-Pro-Tyr-Ile**Ψ[Tz]**Leu	30.0 ± 3.6 (72.0)	n.o.	n.d.
**NT4**	[^177^Lu]Lu-DOTA-PEG_4_-Arg-Arg-Pro-Tyr**Ψ[Tz]**Ile-Leu	46.1. ± 3.8 (164.0)	n.o.	n.d.
**NT5**	[^177^Lu]Lu-DOTA-PEG_4_-Arg-Arg-Pro**Ψ[Tz]**Tyr-Ile-Leu	0 (13.0)	n.o.	n.d.
**NT6**	[^177^Lu]Lu-DOTA-PEG_4_-Arg**Ψ[Tz]**Arg-Pro-Tyr-Ile-Leu	6.5 ± 4.6 (64.9)	6.4 ± 1.2	8.8 ± 1.7
**NT7**	[^177^Lu]Lu-DOTA-PEG_4_**Ψ[Tz]**Arg-Arg-Pro-Tyr-Ile-Leu	2.2. ± 1.2 (46.9)	9.4 ± 0.5	4.5 ± 0.8
**NT8 ^a^**	[^177^Lu]Lu-DOTA-PEG_4_-Arg-Arg-Pro-Tyr-Tle-Leu	70.6. ± 1.4 (n.d.)	1.3 ± 0.2	507 ± 114
**NT9**	[^177^Lu]Lu-DOTA-PEG_4_-Arg**Ψ[Tz]**Arg-Pro-Tyr-Tle-Leu	97.7 ± 2.3 (n.d.)	2.1 ± 0.1	214 ± 45
**NT10**	[^177^Lu]Lu-DOTA-PEG_4_**Ψ[Tz]**Arg-Arg-Pro-Tyr-Tle-Leu	94.7 ± 4.2 (n.d.)	1.2 ± 0.2	>1000
**NT11**	[^177^Lu]Lu-DOTA-PEG_4_**Ψ[Tz]**Arg**Ψ[Tz]**Arg-Pro-Tyr-Ile-Leu	0.2 ± 0.2 (13)	10.8 ± 0.4	4.6 ± 2.3
**NT12**	[^177^Lu]Lu-DOTA-PEG_4_**Ψ[Tz]**Arg**Ψ[Tz]**Arg-Pro-Tyr-Tle-Leu	97.2 ± 3.1 (n.d.)	2.2 ± 0.1	>1000

^a^ Reference compound. ^b^ Determined in blood serum at 37 °C. ^c^ n.d.: not determined ^d^ Ratio of specific receptor-bound and cell-internalised compound expressed in % of administered dose. Expressed as the means of at least two independent experiments. ^e^ Determined by receptor saturation binding assay on HT-29 cells expressing NTR1. Expressed as the means ± SEM of at least two independent experiments.

**Table 7:** Sequences of **CatS1–2** and **CatK1–2** and their reported biological properties [99].

Comp.	Sequence ^a^	K_i_ for CatS, pH 5.5 [nM] ^b^	K_i_ for CatS, pH 7.4 [nM] ^b^	K_i_ for CatK, pH 5.5 [nM] ^b^	K_i_ for CatL, pH 5.5 [nM] ^b^
**CatS1**	Ac-Gly-Arg-Trp-His-Pro-Met-Gly**Ψ[Tz]**Ala-Pro-Trp-Glu-D-Ala-D-Arg-NH_2_	15,000 ± 5000	42,000 ± 8000	10,000 ± 3600	30,000 ± 3200
**CatS2**	Ac-Gly-Arg-Trp-His-Pro-Met-**aza**Gly-Ala-Pro-Trp-Glu-D-Ala-D-Arg-NH_2_	26 ± 5	17 ± 3	3 ± 0.8	5 ± 0.5
**CatK1**	Abz-Arg-Pro-Pro-Gly**Ψ[Tz]**Phe-Ser-Pro-Phe-Arg-Tyr(3-NO_2_)-NH_2_	n.o.	n.o.	800 ± 330	22,000 ± 2000
**CatK2**	Abz-Arg-Pro-Pro-**aza**Gly-Phe-Ser-Pro-Phe-Arg-Tyr(3-NO_2_)-NH_2_	n.o.	n.o.	9 ± 0.7	2000 ± 400

^a^ Abz: *o*-Aminobenzoic acid ^b^ K_i_ values were determined by measuring residual peptidase activity after incubation with the peptidomimetics at 37 °C using Z-Leu-Arg-AMC (Cathepsin S) or Z-Phe-Arg-AMC (Cathepsin K and L) as substrate. Data expressed as the means ± SEM of three individual experiments. n.o.: not observed.

**Table 8:** Sequences of **A7R** and **NV1–3** and their reported biological properties [79].

Compound	Sequence ^b^	Inhibition at 10 μM [%] ^c,d^	IC_50_ [μM] ^d^
**A7R ^a^**	Ala-Thr-Trp-Lys-Pro-Pro-Arg	61.0 ± 0.4	5.86
**NV1**	Lys(Har)-Pro-Ala-Arg ^a^	n.d.	0.3
**NV2**	Lys(Har)-Gly**Ψ[Tz]**Gly**Ψ[Tz]**Arg	58.1 ± 2.1	8.39
**NV3**	d-Lys(Har)-Gly**Ψ[Tz]**Gly**Ψ[Tz]**Arg	52.6 ± 1.3	10.22

^a^ Reference compound. ^b^ Har: Homoarginine ^c^ Expressed as percentage of inhibition of VEGF_165_ binding to NRP-1. Values represent means ± standard deviation (SD). n.d.: not determined. ^d^ Determined in an enzyme-linked immunosorbent assay.

**Table 9:** Sequences of **[Nle^15^]MG11** and **MGN1–15** and their reported biological properties [82,112].

Compound	Sequence	t_1/2_ [h] ^b,d^	Uptake after 4 h [%] ^b,e^	IC_50_ [nM] ^c,f^
**[Nle^15^]MG11 ^a^**	Lu-DOTA-d-Glu-Ala-Tyr-Gly-Trp-Nle-Asp-Phe-NH_2_	3.9 (3.8–4.1)	32.2 ± 3.2	15.4 (11.0–21.1)
**MGN1**	Lu-DOTA-d-Glu-Ala-Tyr-Gly-Trp-Nle-Asp-Phe**Ψ[Tz]**H	34.9 (31.3–39.3)	2.5 ± 2.9	200.5 (164–245)
**MGN2**	Lu-DOTA-d-Glu-Ala-Tyr-Gly-Trp-Nle-Asp**Ψ[Tz]**Phe-NH_2_	35.9 (32.2–40.3)	0.1 ± 0.08	> 50,000
**MGN3**	Lu-DOTA-d-Glu-Ala-Tyr-Gly-Trp-Nle**Ψ[Tz]**Asp-Phe-NH_2_	114.3 (96.5–139.7)	0.2 ± 0.13	> 50,000
**MGN4**	Lu-DOTA-d-Glu-Ala-Tyr-Gly-Trp**Ψ[Tz]**Nle-Asp-Phe-NH_2_	349.8 (263–520)	33.1 ± 1.9	25.4 (18.3–34.6)
**MGN5**	Lu-DOTA-d-Glu-Ala-Tyr-Gly**Ψ[Tz]**Trp-Nle-Asp-Phe-NH_2_	3.8 (3.6–4.0)	41.7 ± 3.9	15.6 (12.3–19.7)
**MGN6**	Lu-DOTA-d-Glu-Ala-Tyr**Ψ[Tz]**Gly-Trp-Nle-Asp-Phe-NH_2_	2.6 (2.5–2.7)	54.3 ± 5.1	1.7 (1.3–2.3)
**MGN7**	Lu-DOTA-d-Glu-Ala**Ψ[Tz]**Tyr-Gly-Trp-Nle-Asp-Phe-NH_2_	51.4 (46.2–57.7)	29.6 ±2.7	20.9 (17.0–25.7)
**MGN8**	Lu-DOTA-d-Glu**Ψ[Tz]**Ala-Tyr-Gly-Trp-Nle-Asp-Phe-NH_2_	7.7 (7.3–8.1)	39.6 ± 2.7	8.0 (6.3–10.9)
**MGN9**	Lu-DOTA-d-Glu-Ala-Tyr**Ψ[Tz]**Gly-Trp**Ψ[Tz]**Nle-Asp-Phe-NH_2_	279.5 (206–431)	28.2 ± 3.0	65.8 (53.2–80.9)
**MGN10**	Lu-DOTA-d-Glu-Ala-Tyr**Ψ[Tz]**Gly**Ψ[Tz]**Trp-Nle-Asp-Phe-NH_2_	1.9 (1.85–2.04)	49.6 ± 3.0	12.4 (10.9–14.0)
**MGN11**	Lu-DOTA-d-Glu-Ala**Ψ[Tz]**Tyr-Gly-Trp**Ψ[Tz]**Nle-Asp-Phe-NH_2_	386.1 (249–842)	31.1 ± 3.9	91.0 (76.3–108.3)
**MGN12**	Lu-DOTA-d-Glu-Ala**Ψ[Tz]**Tyr**Ψ[Tz]**Gly-Trp-Nle-Asp-Phe-NH_2_	4.1 (3.8–4.4)	48.3 ± 2.2	5.3 (4.5–6.1)
**MGN13**	Lu-DOTA-d-Glu**Ψ[Tz]**Ala-Tyr**Ψ[Tz]**Gly-Trp-Nle-Asp-Phe-NH_2_	14.7 (14.0–15.5)	58.4 ± 3.5	5.8 (5.3–6.4)
**MGN14**	Lu-DOTA-d-Glu**Ψ[Tz]**Ala**Ψ[Tz]**Tyr-Gly-Trp-Nle-Asp-Phe-NH_2_	6.4 (5.8–7.2)	40.9 ± 2.5	15.6 (12.6–19.1)
**MGN15**	Lu-DOTA-d-Glu**Ψ[Tz]**Ala**Ψ[Tz]**Tyr**Ψ[Tz]**Gly-Trp-Nle-Asp-Phe-NH_2_	8.1 (7.7–8.6)	47.1 ± 1.6	7.3 (6.0–8.7)

^a^ Reference compound. ^b^ Results obtained with [^177^Lu]Lu-labelled compounds. ^c^ Results obtained with nonradioactive ^nat^Lu-labelled compounds. ^d^ Determined in blood serum at 37 °C. Expressed as means with 95% confidence interval of nonlinear regression of at least two independent experiments. ^e^ Ratio of specific receptor-bound and cell-internalised compound expressed in % of administered dose. Expressed as the means ± SD of at least three independent experiments. ^f^ Determined by receptor saturation binding assay on A431 cells transfected with CCK2R. Expressed as means with 95% confidence interval of nonlinear regression of at least three independent experiments.

**Table 10:** Sequences of **AII** and **AII1–4** and their reported biological properties [115].

Compound	Sequence	IC_50_ for AT2R [nM] ^b^	AT2R/AT1R Selectivity ^c^
**All ^a^**	Asp-Arg-Val-Tyr-Ile-His-Pro-Phe	0.12 ± 0.01	13.7
**[Tyr^6^]All**	Asp-Arg-Val-Tyr-Ile-Tyr-Pro-Phe	4.0 ^d^	18,000 ^d^
**All1**	Asp-Arg-Val-Tyr-Ile**Ψ[Tz]**Tyr-Pro-Phe	1990 ± 88	>5
**All2**	Asp-Arg-Val-Tyr**Ψ[Tz]**Ile-Tyr-Pro-Phe	2.8 ± 0.08	>3611
**All3**	Asp-Arg-Val**Ψ[Tz]**Tyr-Ile-Tyr-Pro-Phe	155 ± 6	>64
**All4**	Asp-Arg**Ψ[Tz]**Val-Tyr-Ile-Tyr-Pro-Phe	7.5 ± 0.05	>1336

^a^ Reference compound. ^b^ Determined in a competition binding assay on HEK-293 cells transfected with AT2R with [^125^I,Sar^1^,Ile^8^]All as competitor. Expressed as means ± SD of three independent experiments. ^c^ Ratio of IC_50_ AT2R/AT1R. ^d^ Values taken from Magnani et al. [114].
